# Clinical efficacy of Delta large-channel endoscopy versus percutaneous endoscopic lumbar discectomy in treating far lateral lumbar disc herniation: a retrospective study

**DOI:** 10.3389/fsurg.2026.1902290

**Published:** 2026-10-13

**Authors:** Yong Xu, Yilei Zhao, Qinyi Jiang, Kangjia Yang

**Affiliations:** Department of Orthopedics, Zhenjiang First People’s Hospital, Zhenjiang, Jiangsu, China

**Keywords:** Delta large-channel endoscopy, far lateral lumbar disc herniation, minimally invasive surgery, percutaneous endoscopic lumbar discectomy, postoperative recovery

## Abstract

**Background:**

Far lateral lumbar disc herniation (FLLDH) accounts for 7%–12% of lumbar disc herniations, causing significant radicular pain, hypoesthesia, and muscle weakness. Due to its lateral location, traditional open surgery has limitations, including extensive tissue disruption and long recovery times. Minimally invasive techniques, such as The Delta large-channel endoscopy and percutaneous endoscopic lumbar discectomy (PELD), have emerged as alternatives, but there is limited comparative evidence on their efficacy for FLLDH.

**Methods:**

This retrospective study included 78 patients with FLLDH, divided into two groups: 39 patients underwent the Delta large-channel endoscopy and 39 underwent PELD. The study compared both procedures in terms of operation time, blood loss, postoperative recovery, pain relief, functional improvement, and complications. All patients had failed conservative treatment and were diagnosed with FLLDH based on MRI and CT scans.

**Results:**

Both surgical methods significantly improved pain and function. The Delta large-channel endoscopy group had longer operation times and more blood loss, but fewer fluoroscopies. Postoperative pain and functional recovery, assessed using VAS and ODI scores, were similar across both groups. The excellent/good rate based on the MacNab criteria was 92.31% for the Delta large-channel endoscopy and 84.62% for PELD, with no significant differences.

**Conclusions:**

Both Delta large-channel endoscopy and PELD effectively relieved pain and improved function in FLLDH patients. The Delta large-channel endoscopy, though requiring longer operation times, showed advantages in early recovery and more thorough decompression for complex cases. The choice of technique should depend on lesion characteristics and surgeon expertise.

## 1 Introduction

Lumbar disc herniation (LDH) is one of the common diseases in spine surgery, which can be divided into central, paracentral, foraminal and extremely lateral according to the protrusion site. Compared with central and paracentral herniation, far lateral lumbar disc herniation (FLLDH) accounts for about 7%–12% of all LDHs, mainly compressing the nerve roots behind the intervertebral foramen, and is clinically manifested by obvious radicular pain, hypoesthesia, and muscle weakness (1–3). Due to the lesion's location on the lateral side of the spinal canal, conventional posterior surgical approaches offer limited visibility. The deep anatomical position and complex adjacent structures (with the pedicle, superior articular process, and nerve root in close proximity) restrict surgical exposure and maneuverability, making treatment particularly challenging (4). Although traditional open surgery (such as lamina fenestration and decompression) can achieve the goal of adequate decompression, it often causes problems such as large bone structure destruction, intraoperative bleeding risk, prolonged postoperative recovery time, and postoperative scarring (5). With the continuous development of minimally invasive spine techniques, various endoscopic surgeries have gradually become one of the main treatment modalities for LDH (6–8). This technique achieves decompression of nerve roots while preserving the stable structure of the spine by establishing a minimally invasive channel, which has the advantages of less trauma, less postoperative pain, and faster recovery (9).

At present, the two most widely used endoscopic procedures are Delta large-channel endoscopy and percutaneous endoscopic lumbar discectomy (PELD). Delta large-channel endoscopy can perform adequate hemostasis, precise decompression, and nerve release through a wide working channel combined with direct endoscopic operation (7, 10, 11). This technique is especially suitable for patients with large herniation areas with bony stenosis or migratory lesions (12). In contrast, PELD enter the lesion space through a percutaneous transforaminal approach, which can avoid spinal canal structures and reduce lamina and articular process damage, especially in cases of foraminal and paracentral herniation (13–15). However, for far lateral herniation, there are still challenges in terms of operating space, microscopic field of view, nerve root protection, etc. Some studies have pointed out that PELD may be limited in managing bony compression, large or highly migrated herniation (10, 16). At present, there are relatively few studies and reports on the comparison of Delta large-channel endoscopy treatment with PELD for far lateral lumbar disc herniation, and most of them are single-center empirical results, and there is a lack of systematic controlled analysis (17).

At present, there is controversy in the treatment of far lateral lumbar disc herniation, and there is no unified standard, and different surgical methods have their own advantages and disadvantages in terms of indications, safety, and thorough decompression. The two surgical procedures require high experience in terms of operating skills, anatomical grasp, and microscopic visual field control, and the operation differences of different doctors may affect the evaluation of efficacy. Therefore, this study intends to retrospectively analyze the patients with far lateral lumbar disc herniation in our hospital, and compare the differences between the two procedures of Delta large-channel endoscopy and PELD in terms of operation time, blood loss, postoperative recovery, pain and functional improvement, complications, etc., through such studies, it can provide a stronger evidence-based basis for clinical surgical selection, and provide guidance in the optimization of surgical procedures and the definition of indications.

## 2 Materials and methods

### 2.1 General data

This study is a single-center retrospective controlled study that included 78 patients with FLLDH treated at our hospital from January 2020 to June 2024. All patients received definitive diagnosis of FLLDH by MRI and CT scans, and underwent surgical intervention after failing to achieve significant symptom relief following standardized conservative treatment for over 3 months. Among the patients, 31 were male and 47 were female; Age 19–82 years old, average (60.72 ± 16.52) years; The duration of the disease was 9–65 months, with an average of (45.15 ± 13.43) months. The distribution of affected segments was: L4/5 in 45 cases and L5/S1 in 33 cases. Patients were retrospectively classified into two groups according to the surgical technique they actually received: the Delta large-channel endoscopy group (*n* = 39) and the PELD group (*n* = 39). Group allocation was not based on randomization or predefined demographic or clinical parameters. The choice of surgical technique was made according to the treating surgeon's clinical judgment, taking into consideration the anatomical characteristics and accessibility of the herniated fragment, the anticipated extent of decompression, and the surgeon's familiarity with the respective technique. There were no significant differences between the two groups in terms of gender, age, disease course and lesion segment (*P* > 0.05), which were comparable. Surgical selection was not determined by a prospective randomization or predefined allocation protocol. The choice between Delta large-channel endoscopy and PELD was made according to the treating surgeon's clinical judgment, taking into consideration the anatomical characteristics and accessibility of the herniated fragment, the anticipated decompression requirements, and the surgeon's familiarity with the respective technique. Therefore, selection bias cannot be completely excluded. This study was approved by the Ethics Committee of Zhenjiang First People's Hospital.

### 2.2 Inclusion and exclusion criteria

Inclusion Criteria: (1) Imaging (MRI and CT) showed that the disc herniation was located outside the intervertebral foramen or behind the intervertebral foramen, compressing the corresponding nerve roots; (2) Single-segment lesion with imaging findings consistent with clinical symptoms, with corresponding nerve root compression symptoms; (3) No significant improvement after at least 3 months of systematic conservative treatment (including bed rest, physical therapy, neurotrophic medication, and epidural blocks when clinically indicated); (4) Single-segment lesion with no concomitant spinal canal stenosis on imaging; (5) Complete follow-up data.

Exclusion Criteria: (1) Central or paracentral disc herniation; (2) Significant concomitant spondylolisthesis, spinal canal stenosis, or spinal instability; (3) Previous surgery at the same lumbar segment; (4) Severe osteoporosis or systemic disease rendering the patient unable to tolerate surgery; (5) Those who are lost to follow-up or have incomplete data after surgery.

### 2.3 Surgical methods

All surgical procedures in both groups were performed by the same senior surgeon, who had 15 years of experience in spinal surgery and endoscopic spinal procedures.

#### 2.3.1 iLESSYS Delta large-channel endoscopic group

Under general anesthesia, the patient was placed in a prone position. The responsible spinal segment was identified and marked using C-arm fluoroscopy. After routine disinfection and sterile draping, a 10-mm longitudinal incision was made along the midline, approximately 10 mm lateral to the marked segment. Sequential dilators were inserted through the incision to reach the intervertebral space. After reconfirmation of the target level under C-arm fluoroscopy, the working channel was gradually expanded, and a surgical working cannula was placed. The iLESSYS Delta interlaminar endoscopic system (joimax® GmbH, Karlsruhe, Germany) was then connected and used for spinal canal decompression under direct endoscopic visualization. Radiofrequency electrodes were used to assist in the exposure of the superior and inferior laminae and part of the ligamentum flavum. Subsequently, microscopic Kerrison rongeurs were used to remove portions of the superior and inferior laminae up to the attachment point of the ligamentum flavum. The hypertrophied ligamentum flavum was excised *en bloc* within the range of the bony decompression to restore adequate dural sac expansion. During the procedure, meticulous manipulation was performed to avoid dural injury. The decompression was then extended contralaterally, removing the contralateral ligamentum flavum and surrounding hyperplastic bone until the dural sac and nerve roots were fully visualized under the endoscope. The dura and nerve roots were protected with a nerve dissector, and the ventral compressive intervertebral disc material was removed to achieve complete decompression. After adequate decompression, the dura was observed to resume pulsation, and the nerve root appeared relaxed. The working channel was then withdrawn, the incision was closed with sutures, and a sterile dressing was applied. The representative case is presented in Figures 1A–L.

**Figure 1:**
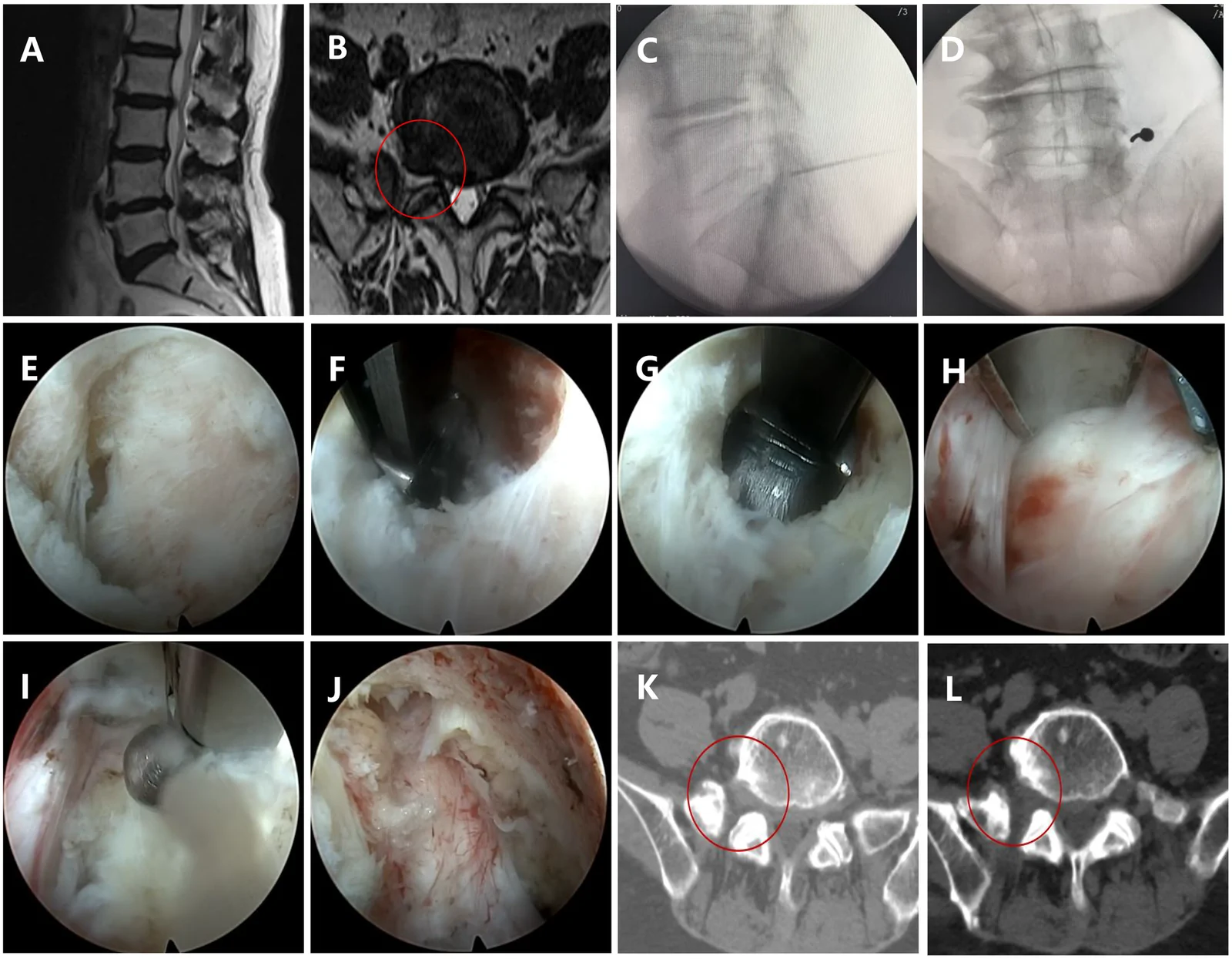
A 77-year-old female patient was diagnosed with L5-S1 far lumbar disc herniation underwent Delta large-channel endoscopic decompression using the iLESSYS Delta interlaminar endoscopic system. **(A,B)** Preoperative magnetic resonance imaging (MRI) showed herniated disc (within the circle). **(C,D)** Intraoperative x-ray anteroposterior and lateral radiographs showed Kirschner's needle positioning; the needle tip was gradually advanced to touch the S1 pedicle isthmus. **(E)** Exposed the lower edge of the L5 transverse process. **(F)** Image showed the lower edge of the L5 transverse process. **(G)** Remove the lateral part of the upper articular process of S1 and the joint capsule. **(H)** Exposed the nerve root exit. **(I)** Removed nucleus pulposus tissue. **(J)** Examination that the nerve root was relaxed. **(K,L)** Preoperative and postoperative CT images showed sufficient decompression in the L5-S1 foramen area. A small amount of bone is removed from the tip of the articular process above S1 (within the circle).

#### 2.3.2 PELD group

The patient was placed in a prone position with a soft pad under the abdomen to reduce intra-abdominal pressure. The responsible intervertebral level and herniated segment were identified using C-arm fluoroscopy, and the puncture site was determined. The puncture point was located at the level of the target intervertebral disc, with a lateral distance of approximately 10.0–12.0 cm from the midline at the L4/5 level and 12.0–14.0 cm at the L5/S1 level. The cephalocaudal angle of the puncture needle was adjusted according to the direction of the herniation. Local infiltrative anesthesia was administered to the skin, deep fascia, and muscle layers. Under anteroposterior and lateral fluoroscopic guidance, an 18-gauge spinal needle was inserted along the preplanned trajectory to the herniated nucleus pulposus site identified on preoperative imaging. In the anteroposterior view, the needle tip was located at the lateral border of the pedicle, and in the lateral view, it was positioned at the posterior–superior region of the intervertebral foramen of the lower vertebra. A guidewire was inserted through the puncture needle, which was then removed. Sequential dilators were placed along the guidewire to gradually expand the soft tissue tract. After adequate dilation, a working cannula was inserted along the guidewire and its position confirmed. The dilators were removed, leaving the working channel in place. For foraminal-type herniations, foraminoplasty was performed when necessary. A 30-degree foraminoscope was introduced through the working cannula. Under direct endoscopic visualization, soft tissue was separated, hemostasis was achieved, and the nerve root and intervertebral disc were identified. Herniated nucleus pulposus tissue was removed using nucleus forceps of various sizes and angles, with assistance from rotation of the working channel when necessary. Once the nerve root was completely decompressed, it appeared freely mobile and floating in the irrigation fluid. Hemostasis was achieved using a radiofrequency electrode, after which the endoscope and working cannula were withdrawn. The skin incision was closed with sutures, and a sterile dressing was applied.

### 2.4 Postoperative management

Postoperatively, patients received routine intravenous fluid therapy, dehydration treatment, and neurotrophic medication. Ambulation was encouraged on the first postoperative day with lumbar brace protection. Patients were advised to avoid bending, heavy lifting, and strenuous activities within one month after surgery. After discharge, all patients were instructed to perform lumbar and back muscle functional exercises. Radiological examinations were conducted regularly during follow-up, which lasted for 12–24 months.

### 2.5 Observation indicators

(1) General surgical indicators: Operation time, intraoperative blood loss, number of fluoroscopies, and length of hospital stay were recorded. (2) Pain and functional scores: Pain intensity of the lower back and legs was assessed using the Visual Analog Scale (VAS, 0–10) preoperatively, and at 1 day, 3 months, and 12 months postoperatively. Lumbar functional recovery was evaluated using the Oswestry Disability Index (ODI, 0%–100%). (3) Evaluation of clinical efficacy: Surgical outcomes were assessed based on the MacNab criteria, categorized as excellent, good, fair, or poor. The excellent and good rates were calculated accordingly. (4) Complications and recurrence: Early postoperative complications, such as nerve root injury, wound hematomat, as well as late recurrence, were documented. Recurrence was defined as the reappearance of radicular symptoms on the same side and at the same level after initial symptom relief, with MRI confirmation of recurrent disc herniation at the same segment.

### 2.6 Statistical analysis

All data underwent statistical analysis via SPSS version 27.0. Measurement data meeting the normal distribution requirement were presented as mean ± standard deviation (*x¯* ± *s*). Intergroup data comparisons were conducted using independent-sample *t*-tests, while intragroup comparisons relied on paired-sample *t*-tests. Categorical variables were examined using the *χ*^2^ test or Fisher's exact test, as appropriate. Because multiple testing of individual complication categories may inflate the familywise type I error rate, the overall complication rate was predefined as a composite safety endpoint and was compared between groups using Fisher's exact test. Individual complications were summarized descriptively without separate hypothesis testing. To analyze the variations in VAS and ODI scores at various time points, repeated-measures analysis of variance (ANOVA) was used. A *P* value of <0.05 was regarded as indicating statistical significance.

## 3 Results

A total of 78 patients with FLLDH were included in this study, with 39 cases in the Delta large-channel endoscopy group and 39 cases in the PELD group. All patients successfully completed the operation, and no cases were converted to open surgery. Both groups were followed up for 12–24 months. There were no statistically significant differences in the general preoperative clinical data between the two groups (*P* > 0.05). The mean age of the Delta large-channel endoscopy group was (60.79 ± 16.42) years, and that of the PELD group was (60.64 ± 16.83) years. The two groups were similar in gender ratio, body mass index (BMI), duration of illness, and affected level (L4/5 and L5/S1). Furthermore, no significant differences were observed between the two groups regarding preoperative VAS scores for leg and back pain, as well as the ODI (Table 1). These outcomes indicated that the preoperative baseline data of the two groups were balanced and had comparability.

**Table 1:** Comparison of general data between the two groups.

Variable	Total (*n* = 78)	Delta large channel endoscopy (*n* = 39)	PELD (*n* = 39)	*P*
Age (years)	60.72 ± 16.52	60.79 ± 16.42	60.64 ± 16.83	0.968
BMI (Kg/m^2^)	22.64 ± 2.88	22.51 ± 2.81	22.76 ± 2.98	0.697
Gender, No. (%)				0.817
Male	31 (39.74)	16 (41.03)	15 (38.46)	
Female	47 (60.26)	23 (58.97)	24 (61.54)	
Affected level, No. (%)				0.492
L4/5	45 (57.69)	21 (53.85)	24 (61.54)	
L5/S1	33 (42.31)	18 (46.15)	15 (38.46)	
Duration of illness (months)	45.15 ± 13.43	44.62 ± 13.53	45.69 ± 13.47	0.726
VAS (LP)	7.44 ± 0.25	7.48 ± 0.26	7.41 ± 0.23	0.217
VAS (BP)	5.51 ± 0.53	5.59 ± 0.50	5.44 ± 0.55	0.2
ODI score	59.70 ± 2.98	59.54 ± 2.49	59.86 ± 3.42	0.635

PELD, percutaneous endoscopic lumbar discectomy; No, number; BMI, body mass index; LP, leg pain; BP, back pain; VAS, visual analogue scale; ODI, Oswestry Disability Index.

Both surgical procedures were successfully completed without serious intraoperative complications. Compared with the PELD group, the operation time of the Delta large-channel endoscopy group was significantly longer, which was (110.82 ± 5.74) min and (80.51 ± 4.82) min (*P* < 0.001), respectively. The intraoperative blood loss was also higher (14.82 ± 1.76) mL, and that of the control group was (7.85 ± 2.93) mL (*P* < 0.001). Despite the increase in operation time and blood loss, the number of intraoperative fluoroscopy times was significantly lower than that of the PELD group, which was (3.36 ± 0.49) and (6.97 ± 0.71) (*P* < 0.001), respectively, suggesting that this procedure was more efficient in intraoperative positioning and operation confirmation (Table 2).

**Table 2:** Comparison of perioperative indicators between the two groups.

Variable	Total (*n* = 78)	Delta large channel endoscopy (*n* = 39)	PELD (*n* = 39)	*P*
Operation time, min	95.67 ± 16.13	110.82 ± 5.74	80.51 ± 4.82	<.001
Intraoperative blood loss (mL)	11.33 ± 4.25	14.82 ± 1.76	7.85 ± 2.93	<.001
Length of hospital stays, day	4.88 ± 1.47	5.08 ± 1.63	4.69 ± 1.28	0.250
Intra-op fluoroscopy, freq	5.17 ± 1.92	3.36 ± 0.49	6.97 ± 0.71	<.001
Total complications	8 (10.27)	2 (5.13%)	6 (15.38%)	0.263
Nerve root injury, No. (%)	3 (3.85)	1 (2.56)	2 (5.13)	1.000
Wound hematoma, No. (%)	2 (2.56)	0 (0.00)	2 (2.56)	0.474
Recurrence, No. (%)	3 (3.85)	1 (2.56)	2 (5.13)	1.000

There was no significant difference in the length of hospital stays between the two groups (*P* = 0.250), which was (5.08 ± 1.63) days and (4.69 ± 1.28) days, respectively. The overall incidence of complications also showed no significant difference between the two groups (*P* = 0.263). In the Delta large-channel endoscopy group, there were 1 case of mild nerve root injury symptom (2.56%), and 1 case of recurrence (2.56%). In the PELD group, there were 2 cases of wound hematoma (5.13%), 2 cases of mild nerve root injury symptom (5.13%), and 2 cases of recurrence (5.13%) (Table 2). All recovered well after conservative treatment. There were no cases of severe neurological deficit or infection in either group. Although the Delta large-channel endoscopic surgery took slightly longer and resulted in slightly more blood loss, the number of fluoroscopies was significantly reduced. In terms of surgical safety, there was no significant difference between the two techniques, both demonstrating good minimally invasive characteristics.

Postoperatively, leg and back pain in both groups were significantly relieved compared with preoperative levels. At 1 day, 3 months, and 12 months after surgery, VAS scores were significantly lower than those before surgery (*P* < 0.001), and ODI scores also significantly decreased (*P* < 0.001). Preoperatively, the Delta large-channel endoscopy group had leg pain VAS of (7.48 ± 0.26), back pain VAS of (5.59 ± 0.50), and ODI of (59.54 ± 2.49), while the PELD group had (7.41 ± 0.23), (5.44 ± 0.55), and (59.86 ± 3.42), respectively, with no significant differences. At 1 day after surgery, the leg pain VAS scores of the two groups decreased to (3.29 ± 0.14) and (3.28 ± 0.14), and the back pain VAS scores decreased to (2.13 ± 0.34) and (2.21 ± 0.41), respectively. At 3 months after surgery, the VAS scores further decreased to (2.33 ± 0.11) and (2.37 ± 0.14), and at 12 months, to (1.25 ± 0.27) and (1.25 ± 0.31), with no significant intergroup differences (*P* > 0.05). The ODI scores also continued to decline, reaching (11.35 ± 2.48) and (10.48 ± 2.37) at 12 months after surgery, with no significant difference between the two groups (*P* = 0.115) (Table 3). Both techniques significantly relieved pain and improved function, and the short- and long-term outcomes were similar.

**Table 3:** Comparison of VAS, ODI score and MacNad evalution between the two groups.

Variable	Total (*n* = 78)	Delta large channel endoscopy (*n* = 39)	PELD (*n* = 39)	Statistic (*t*/*χ*^2^)	*P*	Partial *η*^2^
VAS (LP)						
pre	7.44 ± 0.25	7.48 ± 0.26	7.41 ± 0.23	*t* = 1.25	0.217	
1 day post-	3.28 ± 0.14	3.29 ± 0.14	3.28 ± 0.14	*t* = 0.24	0.811	
3 months post-	2.35 ± 0.12	2.33 ± 0.11	2.37 ± 0.14	*t* = −1.37	0.174	
12 months post-	1.25 ± 0.29	1.25 ± 0.27	1.25 ± 0.31	*t* = 0.00	1.000	
				*F*_(3,74)_ = 9,957.04	<.001	1.00
VAS (BP)						
pre	5.51 ± 0.53	5.59 ± 0.50	5.44 ± 0.55	*t* = 1.29	0.2	
1 day post-	2.17 ± 0.38	2.13 ± 0.34	2.21 ± 0.41	*t* = −0.90	0.369	
3 months post-	1.24 ± 0.43	1.28 ± 0.46	1.21 ± 0.41	*t* = 0.78	0.435	
12 months post-	1.12 ± 0.32	1.13 ± 0.34	1.10 ± 0.31	*t* = 0.35	0.727	
				*F*_(3,74)_ = 1,190.51	<.001	0.98
ODI score						
pre	59.70 ± 2.98	59.54 ± 2.49	59.86 ± 3.42	*t* = −0.48	0.635	
1 day post-	31.98 ± 3.01	32.54 ± 3.07	31.43 ± 2.89	*t* = 1.64	0.105	
3 months post-	26.77 ± 4.17	25.94 ± 4.10	27.60 ± 4.12	*t* = −1.79	0.078	
12 months post-	10.92 ± 2.45	11.35 ± 2.48	10.48 ± 2.37	*t* = 1.60	0.115	
				*F*_(3,74)_ = 3,643.56	<.001	0.99
MacNab evaluation						
Excellent, *n* (%)	31 (39.74)	18 (46.15)	13 (33.33)	*χ*^2^ = 1.34	0.247	
Good, *n* (%)	37 (47.44)	18 (46.15)	19 (48.72)	*χ*^2^ = 0.05	0.821	
Fair, *n* (%)	9 (11.54)	3 (7.69)	6 (15.38)	*χ*^2^ = 0.50	0.478	
Poor, *n* (%)	1 (1.28)	0 (0.00)	1 (2.56)	–	1.000	
Excellence/good rate, *n* (%)	69 (88.46)	36 (92.31)	33 (84.62)	*χ*^2^ = 0.50	0.478	

VAS, visual analogue scale; ODI, Oswestry Disability Index.

According to the MacNab criteria, the excellent and good rate was 92.31% (36/39) in the Delta large-channel endoscopy group and 84.62% (33/39) in the PELD group, with no statistically significant difference between the groups (*P* = 0.478) (Table 3). The 95% confidence intervals for the MacNab excellent/good rate were 79.7%–97.3% in the Delta group and 70.3%–92.8% in the PELD group, respectively. The postoperative clinical symptoms of both groups were significantly improved, with relief of radiating leg pain and improvement in mobility. Most patients were able to resume daily activities and light physical labor within 1 month after surgery. No dural tears occurred in either group. Nerve root injury symptoms in both groups were mild and transient and completely resolved during follow-up. During the 12–24-month follow-up period, there were 1 cases of recurrence (2.56%) in the Delta large-channel endoscopy group and 2 case (5.13%) in the PELD group, with no statistically significant difference (*P* > 0.05). The recurrent cases were effectively treated with secondary minimally invasive surgery, and symptoms were relieved.

Both surgical techniques effectively relieved radicular pain and improved function in patients with FLLDH, but differences remained in surgical efficiency and degree of invasiveness. The Delta large-channel endoscopy group provided a clearer view of the procedure and more thorough nerve root decompression, resulting in faster postoperative pain relief and faster recovery despite longer operative time and more intraoperative bleeding. In contrast, PELD surgery provided a more straightforward approach with less tissue stripping and less bleeding, but with a relatively high rate of postoperative complications and recurrence rates. Both groups showed significant improvements in postoperative VAS, ODI, and excellent rate, but the Delta large-channel endoscopy group was slightly better than the PELD group in terms of early postoperative pain relief and functional recovery. The Delta large -channel endoscopy group is more suitable for most FLLDH patients than PELD as a technique with fast recovery and accurate efficacy. Delta large-channel endoscopy is also suitable for complex cases with bone compression or requiring extensive decompression. The choice of surgical method should be based on the specific comprehensive evaluation of lesion characteristics to obtain the best clinical effect.

## 4 Discussion

This study conducted a retrospective comparative analysis of the clinical outcomes of the Delta large-channel endoscopy and PELD in the treatment of FLLDH. The results showed that both surgical techniques achieved good clinical efficacy in terms of postoperative pain relief and functional improvement. However, differences were observed in perioperative indicators, intraoperative trauma, and early postoperative recovery, with PELD demonstrated advantages in operative efficiency and minimal invasiveness, as reflected by its shorter operative time and lower intraoperative blood loss, whereas Delta large-channel endoscopy required a longer operative time and resulted in greater intraoperative blood loss.

The study found that the operation time of the Delta large-channel endoscopy group was longer than that of the PELD group, and intraoperative blood loss was higher. This may be attributed to the fact that the Delta large-channel endoscopy provides a wider working cannula and a more direct visual field under endoscopy. However, it often requires greater separation of skin and muscles, as well as stepwise dilation to establish a stable working channel. In some cases, limited local bone resection (such as partial removal of the facet margin) is needed to enlarge the surgical field. Therefore, more soft tissue dissection and bone handling are involved, leading to prolonged operation time and slightly increased bleeding. Previous clinical reports on the Delta large-channel endoscopy system have also indicated that when bony obstruction removal or extensive decompression is required, the increased number of surgical steps results in longer operation time and greater blood loss (9, 18–22). In contrast, the minimally invasive advantage of PELD mainly stems from its direct transforaminal approach, which bypasses the posterior lamina and paraspinal muscles, thus avoiding excessive traction and thermal injury. This minimizes surgical trauma, allowing for faster operation and less bleeding, particularly in single-segment, uncomplicated far lateral herniations. Although PELD requires more C-arm fluoroscopy to ensure accurate puncture and channel positioning, the additional radiation exposure is generally added as well. This finding has been repeatedly confirmed in multicenter PELD case series and systematic reviews (23–27). Therefore, the high operation time and bleeding volume observed in the Delta large-channel endoscopy group in this study should be understood as the unavoidable cost of tissue interference in establishing a large operating space. Resecting the posterior yellow ligament and using the rotatory tube to mobilize the nerve root allow direct visualization of the nucleus pulposus, which may facilitate more extensive and controlled decompression, particularly in anatomically complex cases or when bony decompression is required (9, 19).

In this study, both groups showed significant improvement in the medium and long postoperative period with no significant differences between groups. However, the Delta large-channel endoscope demonstrated faster pain relief and functional recovery in the early period (1 day to 1 month postoperatively). This can be attributed to the minimally invasive intervention of the Delta large-channel endoscope on soft tissues and bone structures: more thorough decompression, as well as faster recovery of postoperative activities. Additionally, although both surgical methods require partial bone removal, they do not affect spinal stability, which is beneficial for short-term segmental stability and pain control post-surgery (28, 29). Multiple comparative studies also report that the long-term functional outcomes of the two surgical methods tend to be consistent (14, 24, 30).

The overall complication rates of the two groups in this study were similar. PELD complications are relatively numerous, and due to narrowing of the channel, short-term nerve traction responses or local hemostasis may be more likely to occur when handling bone, and the risk of recurrence may be increased due to residual nucleus pulposus. Therefore, when choosing a surgical approach, it is important not only to compare the overall complication rate but also to evaluate the nature and reversibility of these complications, and to communicate expected outcomes and risks with patients preoperatively (31–33).

Several limitations should be acknowledged. First, this was a retrospective single-center study, which inherently limits the generalizability of the findings. Second, the surgical technique was selected according to clinical judgment rather than random allocation, and selection bias related to lesion morphology, surgeon preference, and temporal trends cannot be completely excluded. Third, although all procedures were performed by the same senior surgeon with 15 years of experience in spinal surgery and endoscopic procedures, the retrospective design and single-surgeon setting may limit the generalizability of our findings to surgeons with different levels of experience. Although the excellent/good rate according to the MacNab criteria was numerically higher in the Delta large-channel endoscopy group than in the PELD group (92.31% vs. 84.62%), the difference was not statistically significant (*P* = 0.478). Given the limited sample size of 39 patients per group, the study may have had insufficient statistical power to detect a modest between-group difference in clinical efficacy. A *post hoc* power analysis based on the observed MacNab excellent/good rates indicated that the statistical power was relatively low (approximately 19%), suggesting a substantial possibility of a type II error. Therefore, the absence of a statistically significant difference should not be interpreted as evidence of equivalence between the two techniques. Rather, the present findings indicate that no significant difference could be detected within the available sample. Larger, adequately powered prospective studies are warranted to determine whether clinically meaningful differences in functional outcomes exist between Delta large-channel endoscopy and PELD.

In the future, a multicenter, prospective randomized controlled study should be carried out and the follow-up cycle should be extended to further verify the long-term efficacy and indication range of the two procedures. In summary, PELD may offer advantages in typical and relatively uncomplicated FLLDH cases because of its shorter operative time and lower intraoperative blood loss. In contrast, Delta large-channel endoscopy may be particularly useful for anatomically complex cases, large or migrated disc fragments, or cases requiring more extensive decompression because of its wider working channel and direct endoscopic visualization. However, the present study did not demonstrate a significant difference in overall clinical outcomes between the two techniques. Therefore, the choice of surgical technique should be individualized according to lesion morphology, anatomical accessibility, the extent of decompression required, and surgeon experience.

## 5 Conclusions

Both Delta large-channel endoscopy and PELD provided significant postoperative improvement in pain and functional status in patients with FLLDH. No significant differences were observed between the two techniques in postoperative VAS or ODI scores or in the overall MacNab outcomes. PELD demonstrated advantages in operative efficiency, with a shorter operative time and lower intraoperative blood loss, whereas Delta large-channel endoscopy required fewer intraoperative fluoroscopic examinations. Given its wider working channel and direct endoscopic visualization, Delta large-channel endoscopy may be useful in anatomically complex cases or when more extensive decompression is required. Therefore, the choice of surgical technique should be individualized according to disc herniation morphology, anatomical accessibility, the extent of decompression required, and surgeon experience. Further multicenter prospective studies with larger sample sizes are warranted to validate these findings.

## Data Availability

The raw data supporting the conclusions of this article will be made available by the authors, without undue reservation.
